# Developing an Educational Resource Aimed at Improving Adolescent Digital Health Literacy: Using Co-Design as Research Methodology

**DOI:** 10.2196/49453

**Published:** 2024-08-07

**Authors:** Callum C Lewis, Melody Taba, Tiffany B Allen, Patrina HY Caldwell, S Rachel Skinner, Melissa Kang, Hamish Henderson, Liam Bray, Madeleine Borthwick, Philippa Collin, Kirsten McCaffery, Karen M Scott

**Affiliations:** 1 Specialty of Child and Adolescent Health, Sydney Medical School, Faculty of Medicine and Health The University of Sydney Westmead Australia; 2 Sydney Health Literacy Lab, School of Public Health, Faculty of Medicine and Health The University of Sydney Sydney Australia; 3 The Children’s Hospital at Westmead Westmead Australia; 4 General Practice Clinical School, Faculty of Medicine and Health The University of Sydney Sydney Australia; 5 Sydney School of Architecture, Design and Planning The University of Sydney Sydney Australia; 6 Institute for Culture and Society Western Sydney University Parramatta Australia

**Keywords:** Adolescent health, digital health literacy, adolescents, online health information, co-design, health education, eHealth literacy, social media

## Abstract

**Background:**

Adolescence is a key developmental period that affects lifelong health and is impacted by adolescents regularly engaging with digital health information. Adolescents need digital health literacy (DHL) to effectively evaluate the quality and credibility of such information, and to navigate an increasingly complex digital health environment. Few educational resources exist to improve DHL, and few have involved adolescents during design. The co-design approach may hold utility through developing interventions with participants as design partners.

**Objective:**

This project aimed to explore the co-design approach in developing an educational resource to improve adolescents’ DHL.

**Methods:**

Adolescents (12-17 years old) attended 4 interactive co-design workshops (June 2021-April 2022). Participant perspectives were gathered on DHL and the design of educational resources to improve it. Data generated were analyzed through content analysis to inform educational resource development.

**Results:**

In total, 27 participants from diverse backgrounds attended the workshops. Insight was gained into participants’ relationship with digital health information, including acceptance of its benefits and relevance, coupled with awareness of misinformation issues, revealing areas of DHL need. Participants provided suggestions for educational resource development that incorporated the most useful aspects of digital formats to develop skills across these domains. The following 4 themes were derived from participant perspectives: ease of access to digital health information, personal and social factors that impacted use, impacts of the plethora of digital information, and anonymity offered by digital sources. Initial participant evaluation of the developed educational resource was largely positive, including useful suggestions for improvement.

**Conclusions:**

Co-design elicited and translated authentic adolescent perspectives and design ideas into a functional educational resource. Insight into adolescents’ DHL needs generated targeted educational resource content, with engaging formats, designs, and storylines. Co-design holds promise as an important and empowering tool for developing interventions to improve adolescents’ DHL.

## Introduction

Adolescence is a foundational developmental period, influencing lifelong well-being through the establishment of lifestyle and behavioral choices [1]. Adolescents may be exposed to many changes and potential risks, including first encounters with sexual behaviors, substance use, and mental illness, and are, hence, a priority group for health promotion [2]. The digital age has transformed adolescence, representing a new landscape for lived experience and challenges in navigating health concerns [3]. Adolescents frequently seek digital health information [4,5], with a vast majority engaging with it on websites and social media [6], often using mobile devices [7].

Digital formats provide a private space for health information seeking, which is particularly beneficial for sensitive health domains, including sexual health [8]. The quantity of digital health information available [9] alleviates information poverty, which was previously encountered by adolescents wanting to access targeted health information [10]. The currency of information [9], alongside the ability to share personal perspectives [11], further promotes uptake.

Despite the frequent use of digital health information by adolescents, awareness of its highly variable quality has engendered a lack of trust [12]. Heightened risks to adolescent health exist due to insufficient regulation of digital health information [13], particularly given poor quality information is frequently disseminated, often driven by commercial interests [14]. This has been accentuated in the current era where misinformation, which adolescents often struggle to identify [15], is frequently published on the internet, including social media, as exemplified by the COVID-19 “infodemic” [16].

Digital health information can impact adolescents’ engagement with health care providers, including decisions about how and when to attend health facilities [17,18], or decisions to self-manage care [19]. Risks due to poor quality information and delayed health care presentations can potentially cause harm [20]. Conversely, opportunity exists to support health engagement if information is accurate and appropriate [9,21]. Given the importance of digital health information, adolescents’ ability to assess its quality needs to be optimized [22].

Digital health literacy (DHL), “the ability to find, evaluate, appraise, integrate, and apply health information from online environments” [23] denotes the multifaceted skills required to assess credibility of digital health information [24]. Adolescents often lack these important digital health literacy skills [25]. In particular, adolescents frequently use ineffective heuristics [26], rather than objective criteria [27], to appraise digital health information, often prioritizing relevance and accessibility over credibility [28,29].

Social cognitive theory (SCT) offers a useful lens through which to analyze DHL. It describes how learning and behavior is influenced by a dynamic interrelationship between a person, their behavior, and their surrounding multifactorial context [30]. Self-efficacy, the notion of individuals trusting their own ability, is a core SCT concept that is understood to be key for effective learning [31], including for building and sustaining DHL skills [32]. However, evidence suggests adolescents frequently overestimate their abilities, perpetuating the impacts of misinformation encountered [27-29]. Education to improve DHL is, therefore, vital, given that higher DHL is associated with positive health behaviors [33], health care engagement, and overall well-being [34,35].

While adolescents’ DHL deficits are increasingly acknowledged, there are few resources that target its improvement, and the literature on resource development is limited. A 2012 trial of a digital classroom resource, which featured instructional DHL content around nutrition and exercise, recommended that intervention designers work more actively with adolescents to ensure relevance, engagement, and efficacy [36]. Self-directed educational resources that are accessible on demand have been deemed beneficial [37], as have digitized formats, due to the engagement and accessibility afforded by these platforms [38].

**Figure 1:**
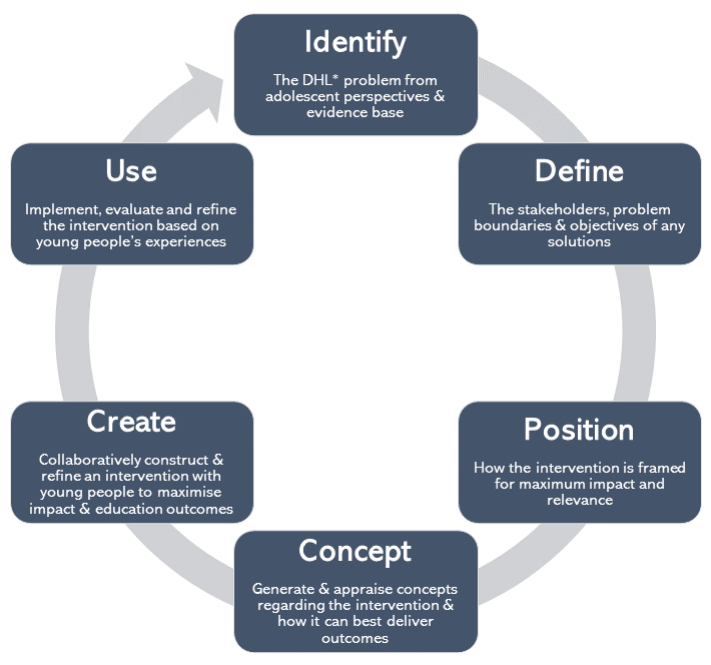
Framework utilized for the co-design of a digital health literacy education resource (after Hagen et al) [42]. DHL: digital health literacy.

Co-design may offer an effective means to produce such educational resources [29]. Avoiding traditional “top-down” [39] approaches, or mere stakeholder consultations, interventions are instead built with the target population [39] as design partners throughout all phases [40]. As a novel method to close “the translational research gap” [41], co-design may improve the relevance and impact of educational resources by ensuring they are consumer driven and consumer centered [42]. Executed using various formats, past trials have used interactive workshops to involve adolescents [43] and articulate research questions into functional products [39], including most recently for mobile app–based mental health interventions [44,45]. Activities can generate creative solutions, through physical “artefacts,” to inform educational resource generation. Evaluation workshops between participants and designers can then ascertain whether the solution effectively responds to participant needs and perspectives, closing the co-design cycle (Figure 1) [42].

Having grown up in the digital age, most adolescents are proficient with common digital technologies, such as the internet and social media, though such proficiency may not be transferred to all forms of technology [26]. Nevertheless, most adolescents are able to effectively express their perspectives on DHL issues and corresponding educational needs [26]. As such, co-design with adolescents holds promise for development of web-based educational resources, despite limited existing research with this participant group [39]. Our study aimed to explore the co-design approach, based on Hagen et al [42] (Figure 1) to develop an adolescent DHL educational resource through a series of interactive workshops. Insight was sought into the influence of co-design on educational resource generation by understanding adolescents’ experience with digital health information and DHL needs, and their design preferences.

## Methods

### Overview

**Figure 2:**
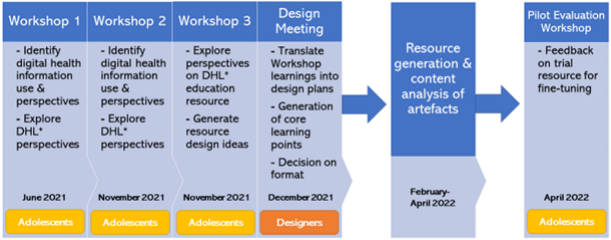
Research design flowchart reflecting the multistage co-design development process of the educational resource. DHL: digital health literacy.

This study involved a qualitative co-design methodology, whereby 4 interactive workshops were held with adolescent participants from June 2021 to April 2022. Data generated were analyzed through inductive content analysis [46] to inform the development of an educational resource with the aim of improving users’ DHL (Figure 2).

### Ethical Considerations

This research project was approved by Sydney Children’s Hospitals Network Human Research Ethics Committee (2020/ETH00578).

The participants, who were minors, gave their informed assent, and their parent or guardian gave their informed consent, to participate in this research study. To protect participant privacy, all study data were deidentified prior to analysis.

Participants were each provided with an AUS $50 gift card (approximately US $35 at the time of the study) for use at a range of stores as compensation for their time.

### Context

To understand adolescent participants' experiences with digital health information, perspectives on DHL issues, and to ascertain their educational needs, 3 co-design workshops were organized. The workshops also sought to generate design solutions for an educational resource that could develop user skills in appraising digital health information. Interactive activities during workshops generated physical artefacts, such as design maps and sketches, which were collected for data analysis (Table 1) [43].

These activities were designed in line with SCT to foster participant collaboration in a safe, peer-based environment [30] in which interactive activities enable learning through the dynamic interrelationship of personal attitudes, behavior, and the environment [30]. The activities align with prior literature calling for the development of DHL interventions through co-design, which encompasses use of practical activities to explore and improve adolescents’ self-efficacy and behavior related to DHL [29]. A design meeting was held among the researchers and the software design team developing the resource to translate workshop findings into educational resource designs. An evaluation workshop with adolescent participants was then conducted to obtain participant feedback on a pilot educational resource. The design meeting and evaluation workshop align with the final phases of the co-design framework, which seek to develop, evaluate, and refine interventions with adolescents (Figure 1) [42].

Co-design facilitators were multidisciplinary academics and research students: a medical educator with experience in eLearning and literacy (KMS), a pediatric clinical academic and child and adolescent health researcher (PHYC), a social scientist focusing on the digital and young people (PC), public health and medical student researchers (MT, CCL, and TBA) and design computing academics and software designers (LB, HH, and MB).

**Table 1:** Methods employed during co-design events, including activities used, artefacts generated and alignment with social cognitive theory.

Co-design event details and purpose and activities			Artefacts generated
**Workshop 1 (June 2021); face-to-face: identified adolescents’ perspectives on their use of digital health information, both web-based and on social media, as well as the concept of digital health literacy**			
	Introduction: adolescent participants annotated a worksheet asking them about the digital health information they use, where they use such resources and reasons for use Social Cognitive Theory (SCT) alignment: Interaction between personal, behavioral, and environmental factors	Annotated worksheets	
	Enablers and barriers: participants collaboratively identified enablers and barriers of digital health information use, wrote them on sticky notes and placed these on a wall. These were then categorized and collated by idea type SCT alignment: Influence of environment on behavior and personal factors	Affinity diagram	
	Credibility rankings: on A4 posters, participants ranked (in order of credibility) 5 digital health information websites SCT alignment: Influence of personal factors on behavior and environment	Annotated or ranked examples	
	Assess and rank: participants assessed the 5 websites using a set of fixed criteria (looks professional, is understandable, I can cross check information) and criteria they generated, and ranked them based on this SCT alignment: Influence of personal factors on behavior and environment	Annotated table	
**Workshop 2 (November 2021); face-to-face: identified adolescents’ perspectives on their use of digital health information, both web based and on social media, as well as the concept of DHL**			
	Misinformation personal stories: participants completed a worksheet with personal examples of people believing misinformation and what made it believable SCT alignment: Influence of environment on behavioral and personal factors	Annotated worksheets	
	Social spaces: participants added color-coded stickers to posters of social media platforms they use regularly, use for fun and use to find information. A set of posters of common platforms (eg, Facebook, TikTok) were preprinted, with participants adding a new poster for any additional platforms they use that were not present SCT alignment: Influence of behavior on personal factors and environment	Sticker-annotated poster	
	Field notes: researchers noted key points of discussion during all exercises	Digital written notes	
**Workshop 3** **(November 2021); face-to-face:** **ascertain perspectives on what an educational resource seeking to improve adolescents’ DHL should include and how it should work to be effective**			
	Brainstorming design ideas: participants identified educational design features and content they believed the design solution should have, wrote them on sticky notes, then added them to a wall of annotated sticky notes. These were then categorized and collated by idea type SCT alignment: Interaction between personal, behavioral, and environmental factors	Affinity diagram	
	Resource design maps: in groups, participants identified the features about form and content that they felt a DHL educational resource should include, then sketched these on posters and labeled them SCT alignment: Interaction between personal, behavioral, and environmental factors	Annotated posters	
	Resource design sketches: participants sketched layout, appearance, format, content, and storylines that they felt would make an effective DHL educational resource SCT alignment: Interaction between personal, behavioral, and environmental factors	Drawings	
	Field notes: researchers noted key points of discussion during all exercises	Digital written notes	
**Design meeting (December 2021); face-to-face:** **translate workshop findings into practical design plans through synthesis with the researchers’ educational requirements of the educational resource**			
	Priority learning points: learning points from the workshops were written on palm cards. Participants (researchers and designers) added a sticker to those they felt were the most important to include in the educational resource. The cards with the most stickers were then prioritized as key learning points SCT alignment: Interaction between personal, behavioral, and environmental factors	Sticker-annotated cards	
	Storyline flowcharts: in groups, participants collaboratively created storylines for the educational resource that they felt would be effective in communicating learning points. These were then drawn as flowcharts on a poster SCT alignment: Interaction between personal, behavioral, and environmental factors	Flowcharts	
	Field notes: researchers noted key points of discussion during all exercises	Digital written notes	
**Pilot Evaluation Workshop (April 2022); virtual: gather participants’ feedback on a trial version of the educational resource, which the design team will use to undertake fine-tuning**			
	Resource trial and field notes: participants were guided through the educational resource app, “*mis-Adventures*,” over a video meeting, enabling them to view and engage with its interactive components. The following question prompts were used to capture perspectives: What did you think about *mis-Adventures*? story, videos decisions or choices made along the way quizzes, explanations expert advice at the end design or look and feel What did you like best about *mis-Adventures*? What did you like least about *mis-Adventures*? How can we improve *mis-Adventures*? Please tell us about any problems you had using *mis-Adventures*. Researchers noted down key responses and points of discussion during the exercise *SCT alignment: Interaction between personal, behavioral, and environmental factors*	Digital written notes	

### Participants

Participants were adolescents aged 12-17 years, representing the high school aged population the educational resource was targeted toward. Recruitment involved advertising on flyers, social media, and through word-of-mouth dissemination. The purposive recruitment strategy sought to capture a heterogenous sample despite a small overall size: we aimed to recruit participants who were of differing ages, genders and cultural backgrounds to enhance the diversity of the perspectives obtained [47], and the broad appeal and relevance of the educational resource. For each workshop, recruitment continued until a quota was met, which was set to facilitate small group sizes to enable interactive and collaborative activities. Participants were free to attend as many workshops as they wished: they were reinvited to any subsequent workshops to increase the continuity of the co-design participation cycle [42], while new participants were also invited to ensure diversity of perspectives.

### Data Collection

**Figure 3:**
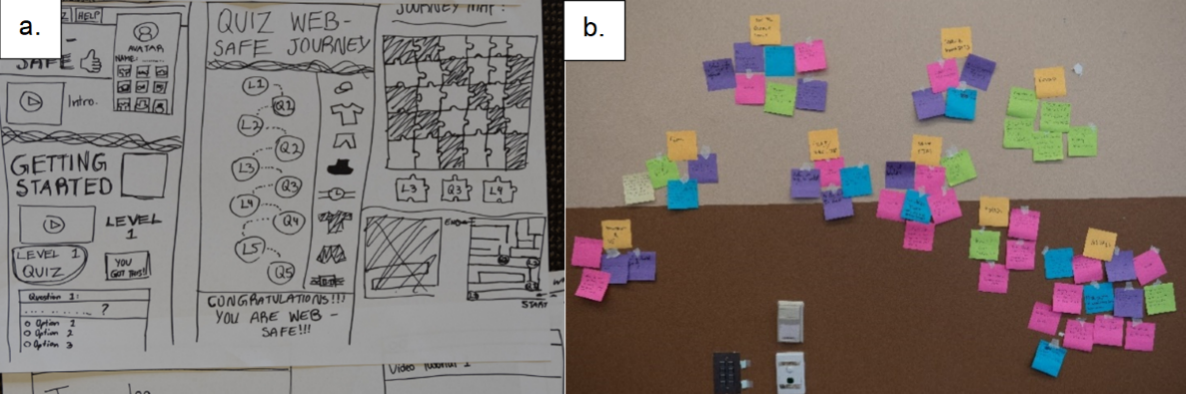
Examples of artefacts obtained from workshops: (A) educational resource design sketches and (B) affinity diagram.

Artefacts collected from the workshops (Table 1) were physical documents, featuring writing and drawings (examples in Figure 3). Artefacts were collected at the completion of each workshop, and subsequently scanned for digitization and analysis.

### Data Analysis

Data analysis involved content analysis of the artefacts generated using NVivo (Release 1.6, QSR International) data management software [48]. This included review and familiarization with the data before iterative coding of the data features in scanned artefacts by CCL using the NVivo pictorial coding tool. An inductive approach was used [46], whereby analysis of the data set informed the codes generated, which comprised recurrent features and ideas in the artefacts. This resulted in the iterative generation of themes, ensuring their relevance to the data [49]. This was completed through an initial categorization of recurrent codes by CCL, with researcher triangulation through analysis meetings with KMS and MT in the first instance, later joined by PHYC, which offered multiple perspectives on coding decisions, enhancing credibility [50]. Through discussion and researcher consensus, similar codes were grouped into concepts and broader categories, which became the themes and subthemes in the data, prior to in-depth analysis and interpretation. Credibility was also developed through obtaining data from multiple workshops with a range of participants.

Results are displayed through a conceptual mind map, generated through *diagrams.net* (JGraph Ltd) software [51].

## Results

In total, 27 adolescents participated in the face-to-face co-design workshops: 7 at workshop 1 and 15 at workshops 2 and 3 (which were held on one day [morning then afternoon] with the same participants), and 5 at workshop 4, the pilot evaluation, which was conducted via web-based platforms because of COVID-19 social distancing restrictions. Participants were of diverse backgrounds, with a mix of genders, ages (12-16 years), and ethnicities (Anglo-Australian, Chinese, Egyptian, Italian, Latin American, Lebanese, and Taiwanese). Seven participants attended multiple workshop days, including 1 participant who attended all 4 workshops.

**Figure 4:**
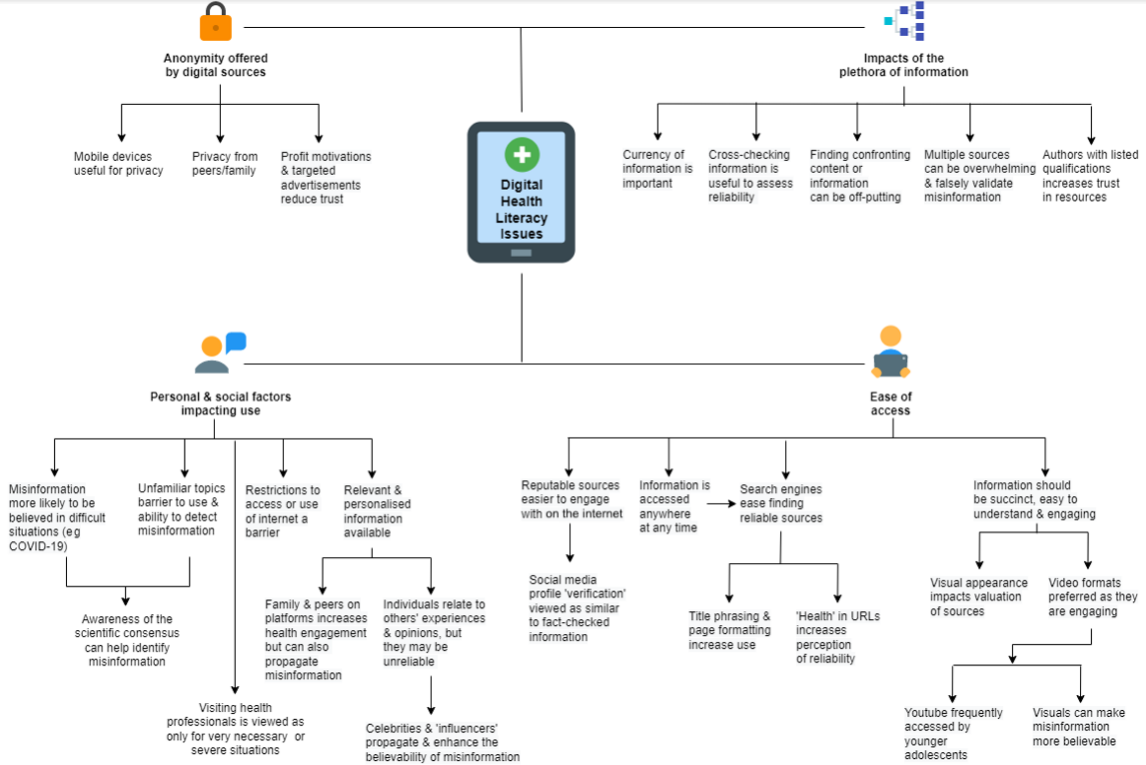
Digital Health Literacy Issues: themes and subthemes representing adolescents’ experiences and issues with digital health literacy.

**Figure 5:**
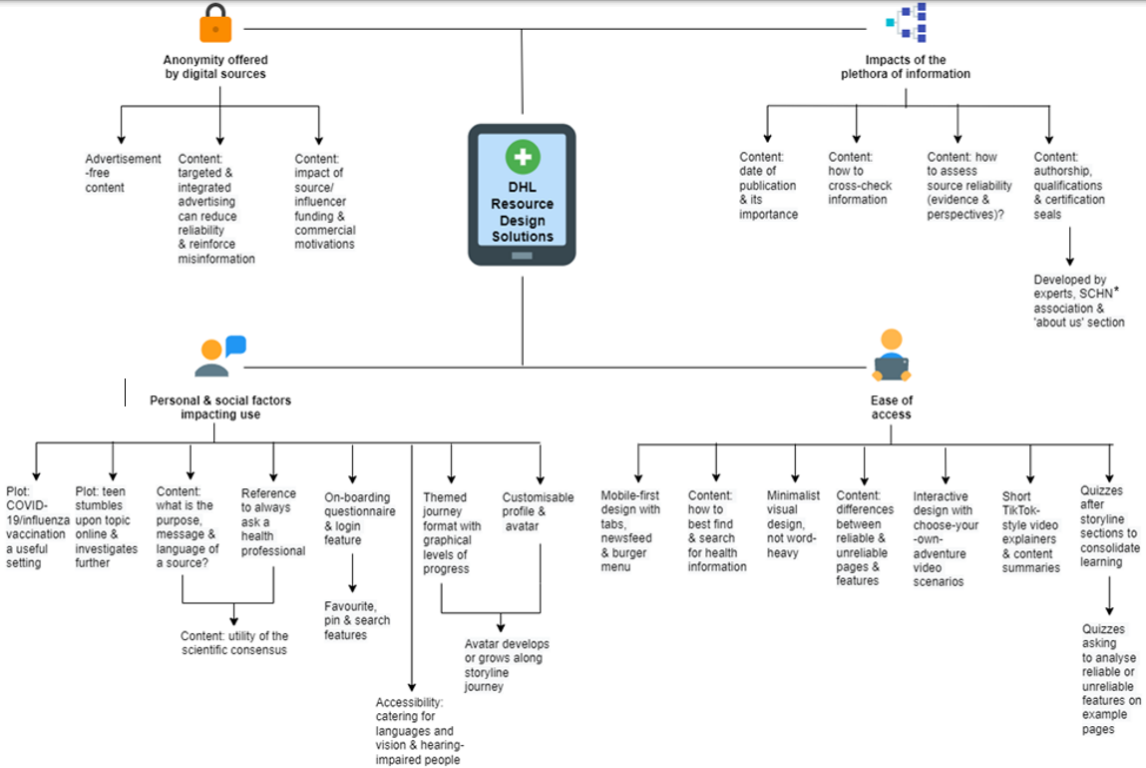
Digital Health Literacy (DHL) Resource Design Solutions: themes and subthemes representing design solutions generated in response to identified DHL needs. SCHN: Sydney Children’s Hospitals Network.

**Figure 6:**
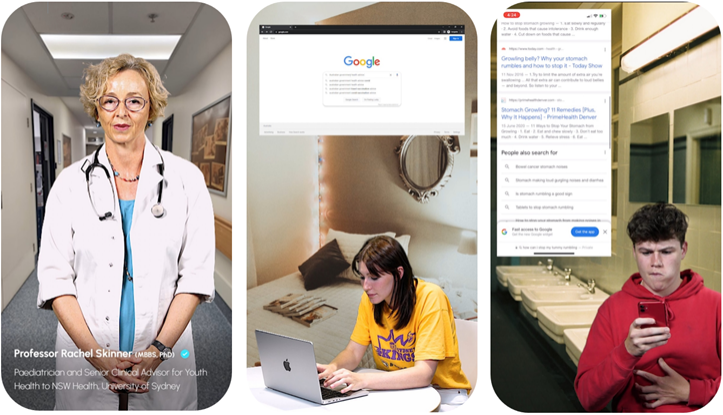
Example screenshots from the digital health literacy resource generated through co-design, featuring health professional explainer videos, realistic digital health information use scenarios, and simulated engagement with digital health information across multiple platforms. The individuals featured in these images have provided their written consent for their image to be published.

Participants’ relationship with digital health information was one of duality—indicating an acceptance of its benefits and relevance in their lives, while having an awareness of its dangers in perpetuating misinformation. This revealed areas of DHL educational need, particularly surrounding appraisal of digital health information quality (Figure 4). Participant-derived educational solutions targeting these issues (Figure 5) highlighted the need for an accessible, self-directed digital resource aligning with participants’ priorities for educational flexibility and autonomy. The educational resource designs that were generated incorporated the most useful aspects of the digital domain to enhance adolescents’ engagement, alongside content that targeted credibility issues at the core of DHL (Figure 6). Through the analysis of these issues and design solutions, we identified 4 key themes: ease of access to digital health information, users’ personal and social factors impacting use of DHL, impacts of the plethora of digital health information available, and anonymity offered by digital sources. These themes are detailed below, each with an analysis of participants’ perspectives and needs, and corresponding design solutions, followed by participant feedback on the pilot educational resource that was created.

### Ease of Access to Digital Health Information

#### User Perspective and Needs

Participants perceived ease of access to digital technology as underpinning their frequent use of digital health information, which satisfied their need for succinct, understandable, and engaging information. They preferred visual, and particularly video, formats due to their engaging nature, with younger adolescents especially accessing video platforms such as *YouTube* and *TikTok* as their first engagement with a social media network. Participants appreciated animated and image-based content for easily communicating concepts. Some perceived that video features increased credibility, even in cases of misinformation, and the aesthetic appearance of platforms was important in determining quality. Participants identified quizzes as an engaging means of understanding and retaining information. Most participants identified mobile devices as a key driver of use, allowing engagement with information across many times and locations, with search engines offering easy access to perceived credible information. Participants indicated that they appraised search results using simple heuristics, including use of website title format and presence of keywords such as “health” in URLs to predict engagement and perception of credibility. They believed the digital domain offered easier engagement with credible sources. This included “verified” profiles (indicated by a blue tick) on social media platforms, which some believed meant content was fact-checked, when at the time of the study it instead signified that the profile belonged to an authentic identity.

#### Co-Designed Educational Solutions

In line with their desires for engaging, highly interactive and concise content, participants envisaged an educational platform of “choose-your-own-adventure-style” stories, featuring branching storylines and plot decision points. This was implemented with content comprised of “*TikTok*-style” videos, which adopted the form’s archetypal features, such as short duration (<3 min), fast pace and satirical tone, adding to the educational resource’s cultural appeal. Videos also featured topic experts explaining DHL concepts and strategies for assessing credibility. As participants desired summaries to consolidate learning, this was enacted using a final video in each storyline, encompassing the educational resource’s cardinal learning points. Catering to participants’ device usage, the user interface has a minimalist “mobile-first” design, featuring a scrollable content carousel and smooth page transitions. Shortcut tabs and drop-down menus linking to specific content afford learning flexibility, with an emphasis on visual features to improve accessibility. Quizzes offer points of revision following videos and include identification of features reflecting credibility on emulated health websites. Automated answers with feedback are provided to enhance their educational benefit. Content includes explanations about the differences between credible and noncredible information, and how to screen search results for credibility.

### Personal and Social Factors Impacting Digital Health Information Use

#### User Perspective and Needs

Participants identified a range of personal and social aspects of adolescent users that affect how they engage with digital health information, including psychological, situational, and peer circumstances. The web and social media–based offering of interactive, personalized information was highly appealing, such as through customization of profiles and content. This included recommended content in newsfeeds, and participants relating to others’ experiences through content sharing and facilities such as forums, messaging features, and comments. This was despite participants often finding such content noncredible, identifying social media “influencers” as a pertinent example of how unqualified individuals can propagate misinformation. Platforms that cater to specific interests and cultural and linguistic groups, such as *WeChat*, which caters for a Chinese population, were popular, along with a preference for educational resources that offer accessibility for the visually and hearing impaired. Participants indicated that family and peers on digital platforms can increase engagement with health information, but also share misinformation. Some found situational factors, such as restrictions to access or content, as a barrier to digital health information use. Conversely, the availability of web and social media–based information meant many only visited health professionals when necessitated by serious situations. Participants indicated that a lack of knowledge and familiarity with specific health topics reduced their engagement and ability to detect incorrect content. For example, they were more likely to trust hyperbolized and inaccurate health content in difficult situations, such as the COVID-19 pandemic. However, participants found that having existing knowledge of the scientific consensus on an issue can aid identification of misinformation.

#### Co-Designed Educational Solutions

The educational resource design was based on storylines featuring a themed journey layout (eg, health preparations before a music festival) to frame users’ progress through it. A live action video format was adopted as a low-resource alternative for participants’ suggestion of an animated avatar with customizable features representing the user. A plot device involved encountering recommended health content on search engine results that leads the character to investigate further, reflecting participants’ experience of often finding health information coincidentally, especially on social media. Captions were developed for video sections to assist users with hearing impairment.

Three storylines, derived from participants, were developed and included in the educational resource. These include a storyline about finding ways to ease gastrointestinal symptoms, based on participant feedback that stories about sensitive issues are compelling. A plotline about deciding whether to get vaccinated against influenza (as an alternative infectious diseases scenario given participants’ interest in COVID-19) was also featured due to participants finding contextually relevant stories engaging. A third storyline involved searching for ways to ease exercise injuries, which was included due to sports injuries being a common target for health products, and its appeal to male demographics.

Educational design solutions targeted difficulties with appraising information about these technical topics through simple explanations about identifying a source’s purpose, message, language, author, date, and provider or funding. A disclaimer—to always seek advice from qualified health professionals—was included in the homepage and throughout the resource due to the potentially significant personal impacts of using digital health information.

### Impacts of the Plethora of Digital Information Available

#### User Perspective and Needs

Participants indicated that the variety and magnitude of health information available on the web and social media impacted engagement. This abundance overwhelmed some or resulted in discouragement if the information was hyperbolic or inflammatory. While accessing information through multiple sources enabled cross-checking, which was believed to be universally important in assessing credibility, it sometimes led to participants falsely verifying sources due to the republishing of misinformation. This included mainstream media reporting on misinformation. Having reputable sources directly discredit such stories was regarded as effective by participants. Participants perceived the presence of author qualifications increased trust in a source, while the currency of information was important for its utility. Transparent referencing and links to further credible information were appreciated.

#### Co-Designed Educational Solutions

Content in the educational resource was based on research into areas of DHL educational need undertaken by the research team, aligning with participant perspectives that authorship by health professionals and educators with listed qualifications and associations with health authorities builds trust. This was coupled with participants’ contributions at co-design workshops, which included their learning priorities and identified areas of DHL deficit, to generate relevant learning content.

Educational design ideas focused on content explaining how to systematically assess source credibility. This included ascertaining whether supporting scientific evidence and multiple perspectives or treatment options are included, the importance of a recent publication date, and how to effectively cross-check information with credible sources. Information about indicators of credible content, such as relevant author health qualifications, was also included. Guided by participants’ suggestions to build trust, the educational resource was developed by experts with listed qualifications and association with well-known health and educational authorities.

### Anonymity Offered by Digital Sources

#### User Perspective and Needs

Participants perceived anonymity when engaging with digital health information to be a core benefit of the digital domain because health topics they viewed were often seen as sensitive or embarrassing. They preferred to use mobile devices because they offered physical privacy from those around them, including peers and family. Participants considered the commercial motivations of web and social media–based publishers as detrimental to credibility, disliking targeted advertisements based on search history as they breached privacy.

#### Co-Designed Educational Solutions

Accordingly, an advertisement-free platform to build trust was implemented. Content about how to identify digital marketing was integrated due to participant interest, including the algorithms and mechanisms behind targeted advertising, and how it exploits users’ confirmation bias to accept false ideas. Content was also included about how integrated health advertising on health information sites can reduce credibility due to conflicts of interest. Content was generated identifying a need to recognize source funding and its impact on information quality, including “influencers” and the commercial partnerships that may underlie their personal stories.

### Educational Resource Feedback

During the pilot evaluation, many participants recognized their designs and stories in the educational resource, which they appreciated, and indicated it made the resource age-appropriate. They found the user interface to be sleek, visually appealing and easy-to-navigate, responding to their desire for a mobile-first platform. Participants also appreciated the fast pace of the video storylines, finding them concise and engaging, enjoying the “choose-your-own-adventure-style” format. For participants, this interactivity was enhanced by the ability to re-explore storylines and alternate subplots by making different decisions. The narrative and character-driven format were enjoyed, especially through the inclusion of fictitious but believable web and social media–based health personalities.

Some users would have liked the educational resource to be compatible with desktop formats, along with the inclusion of accessibility features, such as catering for sensory impairment and featuring different languages. Despite finding the educational resource age appropriate, some participants indicated the quizzes were too easy, suggesting additional levels of complexity for advanced users. This mirrored their proposals for more nuanced storylines that could explore further content with a less predictable plot. Additionally, participants desired more dramatic and engaging videos to convey the plot, including more apparent consequences for making poor decisions based on health information. Some suggested that content summary videos, which feature at the end of each storyline, could be personalized to the user’s in-game decision-making to enhance relevance.

## Discussion

### Principal Findings

The stepwise approach of this study, from initial exploratory workshops to educational design generation, implementation, and evaluation, has enabled the application of the co-design model to the DHL context, yielding a functional and youth-centered product. Co-design elicited authentic adolescent perspectives, encapsulated by the 4 themes regarding digital health information use: ease of access to digital health information, users’ personal and social factors, the plethora of information, and anonymity offered by digital sources. These perspectives encompassed participants’ DHL needs and were addressed through the generation of teaching areas in the educational resource. Targeted educational solutions regarding the most effective modalities and designs were also generated and implemented through integration of participant, researcher, and designer perspectives. The approach enabled the generation of storylines, examples of credible and noncredible sources, and content tone that are highly relevant to an adolescent audience, enhancing potential engagement with and impact of the educational resource.

The results reflect the benefit of co-design in capturing adolescents’ perspectives on their DHL needs, aligning with research into adolescents’ proficiency in this area, despite limited prior engagement in intervention development [26]. Co-design transformed adolescents’ viewpoints into functional educational resource content. In keeping with the importance of social context in Social Cognitive Theory [30] and to influence DHL learning and self-efficacy [32], adolescents’ perspectives were successfully elicited through interactive workshop activities that prompted collaborative social engagement. Ease of access to digital health information, a well-discussed driver of adolescent use [52], underpinned the educational resource’s teaching points, which included identifying noncredible features of information quickly and effectively. Through these teaching points, the educational resource seeks to improve DHL skillsets by targeting the popular but often ineffective heuristics used to assess credibility, such as website names or professional appearance [26,28]. Identification of the personal and social factors affecting digital health information use, and the impact of the diverse information available, generated content that teaches objective appraisal skills that can be universally used [27]. Storylines about sensitive health issues, such as gastrointestinal symptoms, were implemented as they reflect participants’ preference for the anonymity offered by digital platforms, aligning with prior findings [8].

The co-design approach also generated effective modalities and designs for the educational resource for engagement with the adolescent target audience. Ease of access underpinned the DHL needs identified and educational solutions adopted, with strong preference for video as a digital health information format [25]. The educational resource design used these findings as a framework, with short “*TikTok*-style” videos acting as core content within a minimalist mobile-first design, responding to adolescents’ preferred engagement with mobile platforms [7]. Quizzes were incorporated in response to participants suggesting “gamification” [53] features to enhance engagement. In line with Social Cognitive Theory, they may also enhance learning by providing positive feedback to increase user self-efficacy [31]. Similarly, although not implemented, log-in and favorite features, and a themed journey with a customizable avatar, was recurrently suggested by participants, exemplifying how personalization and targeted information brings salience to resources [10].

Co-design enabled the generation of specific storylines and content tone that are highly relevant to the adolescent audience, enhancing potential engagement and impact of the educational resource. The interactive video “choose-your-own- adventure-style” format was key to enabling ease of use, as was the integration of authentic cultural references, such as “viral” social media trends, which were found to be important social factors underpinning engagement. This is mirrored by Paek and Hove’s [36] prior calls for more effective integration of adolescents’ perspectives to enhance the personal relevance and efficacy of educational interventions. Incorporated plot devices, such as a teenager coincidentally encountering a health topic on a social media feed, which leads them to undertake a search, were found by participants to be very common occurrences, paralleling their frequent use of social media to engage with health information [6]. Similarly, subplots regarding “influencers” and sponsored posts were identified and included in the educational resource to add relevance, given their prominence as a DHL issue [14,52]. The capture and implementation of these nuanced references builds upon the use of co-design in different social and cultural contexts, reflecting its versatility [41].

The design meeting, which featured both the research and design teams, was an important aspect of this study’s structure, translating workshop findings into formulated content and development plans through collating and filtering participant ideas. For example, in line with recent literature, COVID-19 was a key suggested topic that exemplified DHL needs [15,23] and was featured in multiple suggested storylines to enhance realism. However, the research and resource development teams implemented an alternative influenza vaccine setting as they believed relying on the current COVID-19 context may reduce educational resource longevity. Although this illustrates a disparity between participant and academic ideology [43], the multistage nature of co-design [39] in this instance generated many other implementable features that embody adolescents’ ideas. The use of interactive collaborative activities to structure the design meeting were in line with the Social Cognitive Theoretical framework and co-design approach [43]. The meeting aligned with prior workshop findings that adolescents perceived reputable sources to be effective at debunking misinformation and wanted expert consensus to shape the educational resource. While this stage of the methods may be incongruous with purist co-design theory, which prioritizes participant involvement during every development phase [42], the impact of COVID-19 lockdowns and project time and resource limitations prevented scheduling of an additional workshop to engage with adolescents at this content development stage.

The feedback obtained through the pilot evaluation workshop was useful in offering an initial indication as to whether the educational resource had adequately responded to the participants’ needs and perspectives. The workshop was largely positive and constructive comments about improvements to the design, plot, and content of the resource led to fine-tuning before finalization. However, time and budget limitations resulted in some of these suggestions being noted but not implemented, presenting areas for further development in subsequent projects. Participants expressed enjoyment in being included in the co-design process, with the final feedback stage important in closing the cycle of participant engagement [42].

### Implications and Recommendations

This study’s co-design approach demonstrates significant translational benefit through the articulation of adolescents’ DHL perspectives and needs (many of which are in line with previous findings [9,26,29,54]) into implemented educational resource solutions [41]. The qualitative methodology held great utility in authentically exploring participants’ ideas in a comfortable, peer-based environment. Mixed methods evaluation of the educational resource is needed to determine the effectiveness of the co-design approach [55].

### Limitations

Although small, the participant sample was heterogeneous. Nevertheless, the sample contained some similar demographics; in particular, all participants were from metropolitan areas as planned recruitment in rural and remote regions was canceled because of social restrictions due to the COVID-19 pandemic [56]. Time limitations due to delays in participant recruitment during COVID-19 lockdowns also prevented participant involvement in storyline plot generation. However, as noted by participants in the pilot evaluation workshop, participants’ design ideas were largely adopted and transformed into a functional product. Time and financial constraints limited the educational resource from featuring avatars, animations, and multiple language options, despite participant suggestions. Proposed social networking features, including sharing, forums, and messaging features, were also excluded as their ongoing moderation was beyond logistical constraints. These may reveal potential directions for future development.

### Conclusions

This study offers novel insights into the implementation and impact of the co-design approach with adolescents to generate DHL educational resources, responding to prior calls for investigation in this area. The workshop format was useful in eliciting authentic adolescent perspectives and enabling their translation to underpin an engaging and functional educational resource that responds to adolescents’ needs. Given these positive initial findings, co-design holds promise as an important tool for developing interventions that could improve DHL in adolescents.

## Data Availability

The data from this study are not publicly available as the participants were minors. The study participants did not provide their informed assent, and their parents and guardians did not provide their informed consent, for these data to be shared. Approval for data sharing was not provided by the Sydney Children’s Hospitals Network Human Research Ethics Committee. Please contact corresponding author Associate Professor Karen Scott (karen.scott@health.nsw.gov.au) for any queries regarding the study data.

## Abbreviations

DHL: digital health literacy
SCT: Social cognitive theory
